# End-to-End Convolutional Neural Network Model to Detect and Localize Myocardial Infarction Using 12-Lead ECG Images without Preprocessing

**DOI:** 10.3390/bioengineering9090430

**Published:** 2022-09-01

**Authors:** Ryunosuke Uchiyama, Yoshifumi Okada, Ryuya Kakizaki, Sekito Tomioka

**Affiliations:** 1 Division of Information and Electronic Engineering, Muroran Institute of Technology, 27-1, Mizumoto-cho, Muroran 050-8585, Hokkaido, Japan; 2 College of Information and Systems, Muroran Institute of Technology, 27-1, Mizumoto-cho, Muroran 050-8585, Hokkaido, Japan

**Keywords:** myocardial infarction, electrocardiogram, 12-lead ECG, convolutional neural network

## Abstract

In recent years, many studies have proposed automatic detection and localization techniques for myocardial infarction (MI) using the 12-lead electrocardiogram (ECG). Most of them applied preprocessing to the ECG signals, e.g., noise removal, trend removal, beat segmentation, and feature selection, followed by model construction and classification based on machine-learning algorithms. The selection and implementation of preprocessing methods require specialized knowledge and experience to handle ECG data. In this paper, we propose an end-to-end convolutional neural network model that detects and localizes MI without such complicated multistep preprocessing. The proposed model executes comprehensive learning for the waveform features of unpreprocessed raw ECG images captured from 12-lead ECG signals. We evaluated the classification performance of the proposed model in two experimental settings: ten-fold cross-validation where ECG images were split randomly, and two-fold cross-validation where ECG images were split into one patient and the other patients. The experimental results demonstrate that the proposed model obtained MI detection accuracies of 99.82% and 93.93% and MI localization accuracies of 99.28% and 69.27% in the first and second settings, respectively. The performance of the proposed method is higher than or comparable to that of existing state-of-the-art methods. Thus, the proposed model is expected to be an effective MI diagnosis tool that can be used in intensive care units and as wearable technology.

## 1. Introduction

Myocardial infarction (MI) is a heart disease that causes necrosis of the myocardium due to obstruction of the coronary arteries [1,2] and is still associated with substantial morbidity and mortality. Necrosis of the myocardium is irreversible; thus, early diagnosis and appropriate treatment of MI are essential. The 12-lead electrocardiogram (ECG), which records cardiac electrical activity from 12 sites on the body, is widely used to diagnose MI [3]. The site of MI is diagnosed by observing and assessing the waveforms of the ECG signal in each lead and combinations of leads exhibiting abnormalities [4,5,6]. Typically, an MI diagnosis by visual observation of a 12-lead ECG requires both significant time and specialized experience.

To date, many research groups have attempted to predict the prognosis from ECG progression in patients with MI [7,8,9]. In these studies, the prognostic prediction was performed using parameter values calculated from ECG signals. However, the appropriate parameters needed to be identified by trial and error based on researchers’ experience and knowledge from a large number of combinations of statistics or feature values. Thus, effective AI-based approaches such as data mining and machine learning are needed in this research area to automatically discover ideal parameters [8].

In contrast, in recent years, many AI-based methods have been proposed to automatically detect and localize MI using ECG data [10,11,12,13,14,15,16,17]. Most of these methods handled ECG data as time-series data and involved complicated preprocessing techniques, e.g., noise reduction, trend removal, beat segmentation, and feature selection. Typically, preprocessing methods involve direct modifications of the ECG data; thus, the performance of the preprocessing methods can directly affect the MI detection and localization performance. In contrast, Jun et al. [18] employed two-dimensional ECG images as the training and testing data to classify arrhythmias using a convolutional neural network (CNN), which is a deep-learning technique. They demonstrated that arrhythmia can be classified with high accuracy without preprocessing, and that the use of ECG images reduced the effect of noise in the ECG signals.

Thus, in this paper, we propose an end-to-end CNN model to detect and localize MI using only ECG images captured from ECG signals without preprocessing. This study was motivated by the work of Jun et al. [18]; however, in the model proposed by Jun et al., the training and classification of the ECG images were performed using only a single-lead ECG signal. However, MI requires the comprehensive assessment of 12-lead ECG signals; thus, it is necessary to extend this model to handle 12-lead ECG images.

Our primary contributions are summarized as follows. First, as in the study by Jun et al. [18], ECG images acquired from ECG signals are used directly as both the training and testing data; thus, we eliminate the need for complicated multistep preprocessing techniques, noise reduction, trend removal, beat segmentation, and feature selection. This simplifies the model, reduces the effect of noise on the ECG signals, and enables quick diagnosis in actual medical practice. Second, the proposed model enables the comprehensive training and classification of 12 sets of ECG images obtained from each lead; thus, diagnoses similar to those of medical professionals can be realized.

The remainder of this paper is organized as follows. Section 2 describes the construction method of the proposed model. Section 3 describes the experimental methodologies, and Section 4 presents the experimental results. Section 5 discusses the observations. Finally, Section 6 concludes the paper, including suggestions for potential future work.

## 2. Materials and Methods

### 2.1. Datasets

In this study, we used 12-lead ECG data from 175 subjects (51 normal subjects and 124 MI patients) collected from the PhysioBank (PTB Diagnostic ECG Database) open access database [19,20]. MI is classified into ST elevation MI (STEMI) and non-ST elevation MI (NSTEMI) based on ECG findings. However, the PhysioBank did not provide information regarding whether the ECG data corresponded to STEMI or NSTEMI. Thus, we collected ECG data for MI as exhaustively as possible without distinguishing between STEMI and NSTEMI. Note that 12-lead ECG data with waveforms deformed by artifacts or those that included arrhythmia were excluded.

### 2.2. Methods

We employed image-converted ECG signals as the training and testing data for the CNN. The proposed method comprises two steps, i.e., (1) ECG image generation, and (2) CNN model construction and classification. Detailed explanations of each step are given in the following.

#### 2.2.1. ECG Image Generation

**Figure 1:**
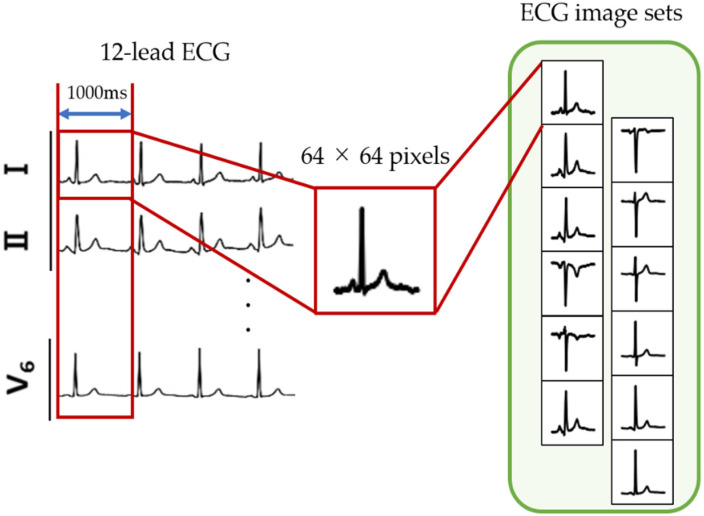
Creation of ECG image sets.

Figure 1 illustrates how ECG images were created from the 12-lead ECG data. First, a subsequence of W milliseconds was taken from the beginning of the ECG data. Next, it was converted to a grayscale image with 256 levels consisting of 64 × 64 pixels in order to express oblique lines and curves smoothly, i.e., to reduce aliasing. This operation was performed on the ECG data acquired by each of the 12 leads. In this paper, the 12 ECG images obtained in this manner are referred to as an ECG image set. This process was repeated via window shifting in increments of W milliseconds toward the terminal direction of the ECG data. Here, W was set to 1000 milliseconds because the average ECG beat in a resting condition is 1 beat/second. The ECG image sets were collected from a normal class and 10 classes of different infarction sites. Table 1 lists the details of the ECG image sets obtained from the 11 classes.

#### 2.2.2. CNN Model Construction and Classification

A CNN is a deep-learning technique that has been used successfully in various tasks, e.g., image recognition and speech recognition [21]. Feature extraction with a CNN is performed by repeating the combination of the convolution layer and pooling layer multiple times [22,23]. The feature maps extracted from the final convolution and pooling layer are converted to a vector and inputted into a fully connected layer to classify the input data [24]. One advantage of CNNs is the high degree of translation invariance, i.e., the ability to identify a particular object in an image even if its position in the image changes [25]. 

**Figure 2:**
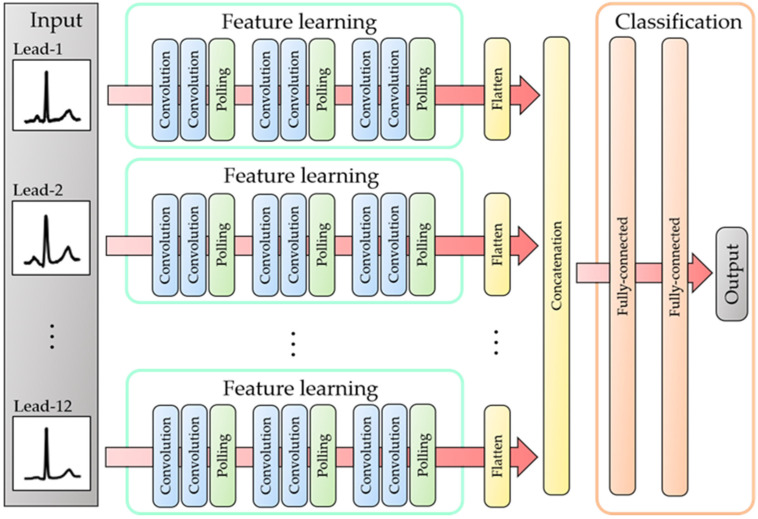
Architecture of proposed CNN model.

The proposed CNN model is unique in that it separately learns the characteristics of the ECG image in each lead using the convolution and pooling layers. Figure 2 and Table 2 show the architecture of the proposed CNN model and the details of the structure, respectively. Here, the input to the proposed model is the ECG image set. First, feature extraction is performed on the ECG image of each lead using the convolution and pooling layers. Then, the features extracted from each lead are unified and inputted into the fully connected layer, which realizes comprehensive learning of the 12-lead ECG images. Finally, the vector output from the fully connected layer is converted to a probability vector using the SoftMax function. In the model-training process, the cross-entropy error is calculated between the probability vector and a one-hot vector corresponding to the true label, and the weights and bias are updated using the backpropagation process. In the model-testing process, i.e., the classification test, the proposed model outputs a class label corresponding to the maximum elements in the probability vector generated from the SoftMax function.

As described in the previous section, ECG images are obtained via window shifting in the ECG signal. Thus, the ECG beats (i.e., waveforms comprising P, Q, R, S, and T waves) are not always fixed at a particular position in the image. However, the proposed CNN model allows us to capture the features of the beats at different locations according to the translation invariance in the CNN.

## 3. Experiments

### 3.1. Experimental Setup

In this study, cross-validation experiments were conducted to evaluate the classification performance of the proposed CNN model. These experiments were performed under the following two settings.

- Setting 1:

In the first experiment, ten-fold cross-validation was conducted, where the ECG image sets of each class were divided equally and randomly. In this setting, we allowed the ECG image sets derived from the same subject (i.e., a normal subject or patient) to be included in both the training and testing data. Note that this is the experimental setting considered in most previous studies.

- Setting 2:

In the second experiment, two-fold cross-validation was conducted, where the data for one patient were used for testing, and the data for all other subjects were used for training. Liu et al. stated that a classifier based on fixed training data may misclassify new patients because ECG data frequently exhibit different characteristics depending on the patients [26]. In fact, it is extremely rare that the ECG data of a patient to be diagnosed exist in the training data. Thus, this experimental setting allowed us to evaluate the performance from a practical perspective. 

In Setting 1, we performed binary classification of the normal and MI class, as well as multiclass classification of the normal class and the 10 classes of different infarction sites. In Setting 2, it was necessary to divide the ECG image sets for each patient. Thus, we excluded the class with only one patient and used the remaining eight classes (i.e., the normal class and seven infarction site classes).

### 3.2. Evaluation Indices

The classification performance was evaluated using the following indices.

(1) $$Sensitivity=\frac{TP}{TP+FN},$$

(2) $$Specificity=\frac{TN}{TN+FP},$$

(3) $$Accuracy=\frac{TP+TN}{TP+FP+TN+FN},$$

Here, TP TN, FP, and FN indicate the number of true positives, true negatives, false positives, and false negatives, respectively.

## 4. Experimental Results

### 4.1. Classification Results for Setting 1

#### 4.1.1. MI Detection Results

Table 3 shows the confusion matrix obtained via binary classification between the normal class and the MI class, and Table 4 shows the scores for each index calculated from the classification results. As can be seen, binary classification was achieved with high accuracy. These results demonstrate that there were clear differences in the features of the ECG images between the two classes and that the proposed CNN model could extract features that were effective for the discrimination of the two classes from the ECG images of each lead.

#### 4.1.2. MI Localization Results

Table 5 summarizes the confusion matrix for the MI localization and classification accuracy for each class. As can be seen, all infarction sites and the normal class were classified with high accuracy. However, the misclassification percentage of the normal class increased compared to the binary classification. In addition, misclassifications were scattered among different infarction sites, especially anterior wall infarction (A, AL, and AS) and inferior wall infarction (I and IL). These misclassifications were due to the fact that the decision boundary became complicated by the increased number of classes.

### 4.2. Classification Results for Setting 2

#### 4.2.1. MI Detection Results

Table 6 shows the confusion matrix obtained by the binary classification between the normal and MI classes, and Table 7 shows the scores for each index calculated from the classification results. Compared to the results obtained in Setting 1, there was a significant decrease in the specificity score, i.e., in many cases, a normal ECG was misclassified as the MI class. This may have been due to the imbalance in the class distribution within the training data. In fact, the number of ECG image sets in all the MI classes was approximately four times greater than that of the normal class.

#### 4.2.2. MI Localization Results

Table 8 summarizes the confusion matrix for the MI localization and the classification accuracy for each class. As can be seen, the overall accuracy was 0.6927, which is significantly less than that of Setting 1. This indicates that the characteristics of the ECG signals differ more or less between different patients (even for the same infarction site).

## 5. Discussion

### 5.1. Results of Setting 1

**Figure 3:**
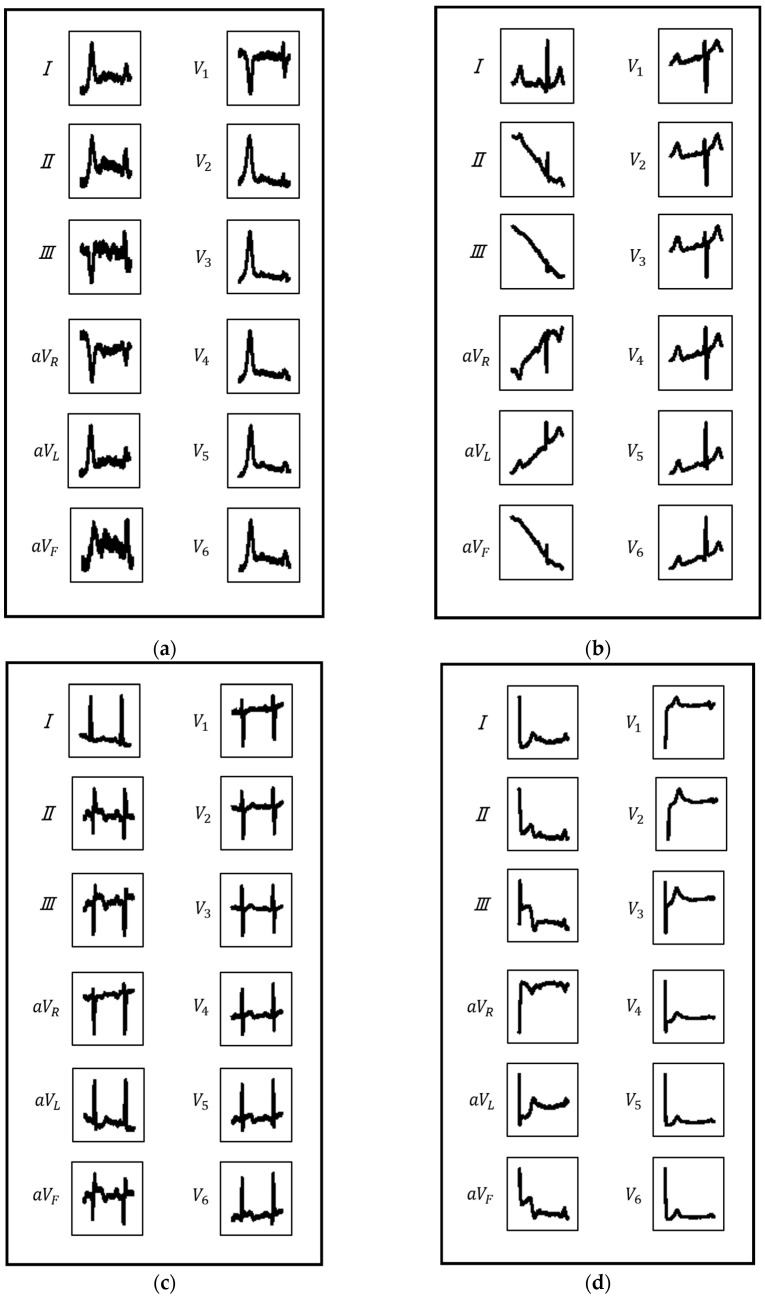
Misclassified ECG waveforms: (**a**) ECG waveform with strong noise; (**b**) ECG waveform with strong trend; (**c**) ECG waveform with two beats; and (**d**) ECG waveform with most of the beat missing.

As shown in Table 9, the proposed model exhibited performance that is comparable to that of state-of-the-art methods. Note that the proposed method obtained such results despite the simple approach of learning ECG images obtained via window shifting. However, the proposed model also demonstrated a serious drawback. The proposed method extracts ECG images by shifting from the start to the end of the ECG signal using a fixed window width. Thus, these images may contain ECG waveforms exhibiting both the characteristics of individual classes and unclear characteristics. Such ECG waveforms can have a negative impact on the model’s training and testing processes. Therefore, we investigated ECG waveforms that caused misclassification, and we found that misclassified ECG waveforms fall into four major patterns: (1) ECG waveforms with strong noise; (2) ECG waveforms with strong trend; (3) ECG waveforms with two beats; and (4) ECG waveforms with most of the beat missing. Each pattern is illustrated in Figure 3a–d. First, we discuss Figure 3a,b. We expected that the influence of noise and trends could be reduced by imaging the ECG signal with a smaller window width; however, in reality, the ECG images with strong noise and trends were generated, and such ECG images can cause a reduction in classification performance. A possible solution to this problem is to incorporate residual blocks [27] and an attention mechanism [28] into the model. This would enhance the noise-reduction performance of the model [29] and realize the dynamic identification of waveform regions to focus on. Thus, the noise and trend in the ECG signal can be reduced. Next, we discuss Figure 3c,d. In this study, the window width was fixed at 1000 milliseconds; thus, individual differences in heart rate could cause duplicating and missing waveforms in a single ECG image. Such ECG images also cause a reduction in classification performance. This problem can be addressed by calculating the heart rate when imaging the ECG signal and dynamically switching the window width depending on the individual patient.

### 5.2. Results of Setting 2

In Setting 1, we allowed ECG images derived from the same subject to be included in both the training and testing data. Thus, as shown in Table 9, many existing methods (including the model proposed in this paper) have obtained very high classification accuracy. However, in Setting 2, the subjects used in the training and testing data were separated completely; thus, classification was more difficult in Setting 2 than in Setting 1. To date, several studies have conducted experiments similar to Setting 2 of this study. Table 10 compares the results of the current study and those of previous studies based on deep-learning algorithms. As can be seen, the scores are quite low for all techniques compared those of Setting 1 in this study. Typically, ECG abnormalities in MI vary according to the location of the infarction and various other factors, e.g., individual differences, progression, and the measurement environment. Thus, it is difficult to comprehensively learn the characteristics of the ECG waveform for all MI types. In the medical field, data from patients not included in the training data will be inputted; thus, it is necessary to increase the training data of MI patients as much as possible to construct a model with sufficiently high generalizability.

Table 10 shows that the proposed model obtained the highest sensitivity score in terms of MI detection. This means that the proposed model identified MI most accurately among all the compared methods. However, the specificity of the proposed model was considerably less than that of the other methods. This means that the proposed model misclassified normal ECGs as MI classes in more cases than the compared methods. This was due to the imbalance in the class distribution within the training data, as mentioned in Section 4.2.1. In addition, this imbalance increases the likelihood that the normal ECG images with unclear characteristics, e.g., those shown in Section 5.1, will be misclassified as the MI class. This problem can be addressed by increasing the number of normal ECG images to the same extent as that of the MI class. 

In the MI localization task, the proposed model obtained the highest scores for all indices. The existing methods employed five classes of the infarction sites, whereas seven classes of infarction sites were considered in the current study. Note that the proposed model demonstrated more accurate results despite the use of additional classes. In addition, the proposed model obtained state-of-the-art performance even when raw ECG images without preprocessing were used as both the training and testing data.

### 5.3. Study Advantages and Limitations

The primary advantages of the proposed model are summarized as follows.

- The proposed model does not require complicated preprocessing, e.g., noise reduction, trend removal, beat segmentation, and feature selection.

- With the proposed model, it is possible to detect and localize MI by comprehensively checking the characteristics of the ECG images for each lead (similar to the diagnoses of medical professionals).

The primary limitations of the proposed model are summarized as follows.

- It is possible to misclassify ECG images with extremely strong noise and trends.

- It is possible to misclassify ECG images with multiple beats or ECG images with most of the beats missing.

### 5.4. Discussion for Practical Application of the Model

Here, we discuss important issues to be addressed and validated for the practical application of the model in the medical field. There are three main issues. The first issue concerns the number of subjects used in the proposed model. In this study, we collected ECG data of MI patients for each infarction site as exhaustively as possible from the PhysioBank database. However, for some infarction sites, ECG data from only one or two patients were used as shown in Table 1. This is not a sufficient number of patients. Thus, it may lead to overfitting in the model by using only biased cases as well as poor classification performance due to the class imbalance problem. To improve the generalization ability of the model, the number of subjects in such minor classes needs to be further increased. The second issue concerns the explainability of the model. The proposed model outputs class labels as classification results, but cannot present information regarding diagnostic rationales, such as abnormal ECG regions that contributed to the classification. The diagnostic rationales output from the model would be important information to support the physician’s subsequent decision making. This issue can be solved using visualization techniques such as Grad-CAM [32], which can display image areas that contribute to classification. In addition, it is important to present scores based on ECG indices as proposed in the previous research [7,8,9,33]. Such scores can provide important information regarding fast triage and prognostic effects for MI patients. The third issue concerns model complexity. In general, neural network models require a large number of computational resources to process a huge number of parameters. Therefore, the use of high-performance GPU computers is essential. To realize a wide range of uses in medical practice, it is necessary to construct a simpler model that can even work on small-scale electronic devices such as smartphones and wearable terminals. To address this issue, model-compression techniques [34,35] would be an effective approach.

## 6. Conclusions

In this paper, we proposed an end-to-end CNN model to detect and localize MI using 12-lead ECG images captured via window shifting from ECG signals without complicated preprocessing. We found that the proposed model demonstrated a classification performance that is higher than or comparable to that of existing state-of-the-art methods. Thus, we consider that the proposed model can be used as an effective MI diagnosis tool in medical practice. 

In the future, we plan to solve the two limitations stated in Section 5.3 and improve the model for practical use as discussed in Section 5.4. AI-based techniques will be able to provide scoring systems in patients with MI similar to diastolic heart failure and other diseases. We believe that a scoring system implementing an accurate MI classifier such as the proposed model would be a powerful tool for physicians to support rapid diagnosis, triage, and prognosis prediction.

## Figures and Tables

**Table 1:** Details of ECG image set for each class.

Class (Abbreviation)	Number of Subjects	Number of ECG Data	Number of ECG Image Sets
Normal (N)	51	74	4837
Anterior (A)	17	47	2812
Anterior–Lateral (AL)	14	39	2580
Anterior–Septal (AS)	27	77	4620
Inferior (I)	30	87	5268
Inferior–Lateral (IL)	23	55	3315
Inferior–Posterior (IP)	1	1	38
Inferior–Posterior–Lateral (IPL)	8	19	1118
Lateral (L)	1	3	180
Posterior (P)	1	4	240
Posterior–Lateral (PL)	2	5	300
Total	175	411	25,308

**Table 2:** Details of the structure of proposed CNN model.

Layer	Number of Input Nodes	Number of ECG Output Nodes	Kernel Size	Batch Normalization	Activation Function
Convolution 1	$64^{2}$	$4\times 62^{2}$	3×3	True	ReLU
Convolution 2	$4\times 62^{2}$	$8\times 60^{2}$	3×3	Ture	ReLU
Pooling 1	$8\times 60^{2}$	$8\times 29^{2}$	4×4	False	-
Convolution 3	$8\times 29^{2}$	$16\times 27^{2}$	3×3	True	ReLU
Convolution 4	$16\times 27^{2}$	$16\times 25^{2}$	3×3	True	ReLU
Pooling 2	$16\times 25^{2}$	$16\times 12^{2}$	3×3	False	-
Convolution 5	$16\times 12^{2}$	$32\times 10^{2}$	3×3	True	ReLU
Convolution 6	$32\times 10^{2}$	$32\times 8^{2}$	3×3	True	ReLU
Pooling 3	$32\times 8^{2}$	$32\times 3^{2}$	4×4	False	-
The flattened vectors of the 12 leads are concatenated					
Fully connected 1	3456	2048	-	True	ReLU
Fully connected 2	2048	1024	-	True	ReLU
Fully connected 3	1024	11	-	False	SoftMax
Loss function	Cross-entropy loss				
Optimizer	Adam				

**Table 3:** Confusion matrix for normal and MI classes in Setting 1.

		Predicted Class	
		N	MI
**True Class**	**N**	4822	15
	**MI**	31	20,440

**Table 4:** Classification performance of normal and MI classes in Setting 1.

Index	Score
Sensitivity	0.9985
Specificity	0.9969
Accuracy	0.9982

**Table 5:** Confusion matrix for MI localization and classification accuracy for each class in Setting 1.

		Predicted Class											
		N	A	AL	AS	I	IL	IP	IPL	L	P	PL	Accuracy
**True class**	**N**	4818	1	0	5	13	0	0	0	0	0	0	0.9961
	**A**	2	2782	3	15	7	3	0	0	0	0	0	0.9893
	**AL**	3	5	2551	17	2	2	0	0	0	0	0	0.9888
	**AS**	3	11	4	4590	9	1	0	1	0	1	0	0.9935
	**I**	6	5	0	5	5243	6	0	2	1	0	0	0.9953
	**IL**	1	1	3	1	14	3286	0	9	0	0	0	0.9913
	**IP**	0	0	0	0	0	0	38	0	0	0	0	1.0000
	**IPL**	0	0	1	1	4	6	0	1106	0	0	0	0.9893
	**L**	0	0	0	0	0	0	0	0	180	0	0	1.0000
	**P**	1	0	0	1	2	0	0	0	0	236	0	0.9833
	**PL**	0	0	0	1	2	0	0	0	0	0	297	0.9900
	**Total**												0.9928

**Table 6:** Confusion matrix for normal and MI classes in Setting 2.

		Predicted Class	
		N	MI
**True Class**	**N**	3718	1119
	**MI**	390	19,623

**Table 7:** Classification performance of normal and MI classes in Setting 2.

Index	Score
Sensitivity	0.9805
Specificity	0.7687
Accuracy	0.9393

**Table 8:** Confusion matrix for MI localization and classification accuracy for each class in Setting 2.

		Predicted Class								
		N	A	AL	AS	I	IL	IPL	PL	Accuracy
**True class**	**N**	4012	24	21	85	515	132	26	22	0.8294
	**A**	91	1524	646	329	177	45	0	0	0.5420
	**AL**	79	311	1692	329	152	16	1	0	0.6558
	**AS**	156	140	731	3239	207	145	1	1	0.7011
	**I**	471	158	167	75	3876	429	90	2	0.7358
	**IL**	118	64	10	113	790	2098	92	30	0.6329
	**IPL**	16	3	8	0	190	256	622	23	0.5564
	**PL**	13	0	9	4	59	59	6	150	0.5000
	**Total**									0.6927

**Table 9:** Comparison of classification performance between proposed model and existing methods under Setting 1.

Author (Year)	Methods	MI Detection Results	MI Localization Results
Arif et al., 2012 [10]	k-NN	Sensitivity = 99.97%	Accuracy = 98.8%
		Specificity = 99.9%	
Safdarian et al., 2014 [11]	• Probabilistic Neural Network (PNN)	Accuracy = 94%	Accuracy = 76%
	• k-NN		
	• Multilayer Perceptron (MLP)		
	• Naive Bayes Classification		
Sharma et al., 2015 [12]	• SVM-Lin	Accuracy = 96%	Accuracy = 99.58%
	• SVM-RBF	Sensitivity = 93%	
	• k-NN	Specificity = 99%	
Acharya et al., 2016 [13]	k-NN	Accuracy = 98.8%	Accuracy = 98.74%
		Sensitivity = 99.45%	Sensitivity = 99.55%
		Specificity = 96.27%	Specificity = 99.16%
Baloglu et al., 2019 [14]	Deep CNN	N/A	Accuracy = 99.78%
Sugimoto et al., 2019 [15]	• Convolutional autoencoder • k-NN	Accuracy = 99.87%	Accuracy = 99.88%
		Sensitivity = 99.91%	Sensitivity = 99.12%
		Specificity = 99.59%	Specificity = 99.92%
Cao et al., 2022 [17]	• SENet • Grad-CAM	Accuracy = 99.98%	Accuracy = 99.79%
		Sensitivity = 99.94%	Sensitivity = 99.88%
		Specificity = 99.94%	Specificity = 99.98%
Proposed model	CNN	Accuracy = 99.82%	Accuracy = 99.28%
		Sensitivity = 99.85%	Sensitivity = 99.21%
		Specificity = 99.69%	Specificity = 99.61%

**Table 10:** Comparison of classification performance between our method and the existing methods under Setting 2.

Author (Year)	Methods	MI Detection Results	MI Localization Results
Fu et al., 2020 [30]	MLA-CNN-BiGRU	Accuracy = 96.50%	Accuracy = 62.94%
		Sensitivity = 97.10%	Sensitivity = 63.97%
		Specificity = 93.34%	Specificity = 63.00%
Han et al., 2020 [31]	ML-ResNet	Accuracy = 95.49%	Accuracy = 55.74%
		Sensitivity = 94.85%	Sensitivity = 47.58%
		Specificity = 97.37%	Specificity = 55.37%
Proposed model	CNN	Accuracy = 93.93%	Accuracy = 69.27%
		Sensitivity = 98.05%	Sensitivity = 65.96%
		Specificity = 76.87%	Specificity = 82.94%

## Data Availability

Not applicable.

## References

[1] National Heart, Lung, and Blood Institute What Is a Heart Attack?. https://www.nhlbi.nih.gov/health/heart-attack.

[2] Guyton A.C., Hall J.E. (2006). Textbook of Medical Physiology.

[3] Thygesen K., Alpert J.S., Jaffe A.S., Simoons M.J., Chaitman B.R., White H.D., Bax J.J., Baumgartner H., Ceconi C., Dean V. (2012). Third universal definition of myocardial infarction. Circulation.

[4] Timmis A.D. (1994). Will serum enzymes and other proteins find a clinical application in the early diagnosis of myocardial infarction?. Br. Heart J..

[5] Lewis K.M., Handal K.A. (2000). Sensible Analysis of the 12 Lead ECG.

[6] Timmis A.D. (1990). Early diagnosis of acute myocardial infarction. Br. Med. J..

[7] Meloni L., Marchetti M.F., Cacace C., Congia M., Scotto R., Caddeo P., Montisci R. (2018). Prognosis and first diagnostic ECG in STEMI patients referred to the emergency medical system for primary PCI. J. Electrocardiol..

[8] Hayıroğlu M.İ., Türkkan C., Tekkeşin A.İ. (2020). Ideal admission electrocardiographic parameters in STEMI: What else do we need to learn?. J. Electrocardiol..

[9] Hayıroğlu M.İ., Lakhani I., Tse G., Çınar T., Çinier G., Tekkeşin A.İ. (2020). In-hospital prognostic value of electrocardiographic parameters other than ST-segment changes in acute myocardial infarction: Literature review and future perspectives. Heart. Lung. Circ..

[10] Arif M., Malagore I.A., Afsar F.A. (2012). Detection and localization of myocardial infarction using K-nearest neighbor classifier. J. Med. Syst..

[11] Safdarian N., Dabanloo N.J., Attarodi G. (2014). A new pattern recognition method for detection and localization of myocardial infarction using T-wave integral and total integral as extracted feature from one cycle of ECG signal. J. Biomed. Sci. Eng..

[12] Sharma L.N., Tripathy R.K., Dandapat S. (2015). Multiscale Energy and eigenspace approach to detection and localization of myocardial infarction. IEEE Trans. Biomed. Eng..

[13] Acharya U.R., Fujita H., Oh S.L., Hagiwara Y., Tan J.H., Adam M. (2016). Automated Detection and localization of myocardial infarction using electrocardiogram: A comparative study of different leads. Knowl. Based. Syst..

[14] Baloglu U.B., Talo M., Yildirim O., Tan R.S., Acharya U.R. (2019). Classification of myocardial infarction with multi-lead ECG signals and deep CNN. Pattern. Recognit. Lett..

[15] Sugimoto K., Kon Y., Lee S., Okada Y. (2019). Detection and localization of myocardial infarction based on a convolutional autoencoder. Knowl. Based Syst..

[16] Acharya U.R., Fujita H., Oh S.L., Hagiwara Y., Tan J.H., Adam M. (2017). Application of deep convolutional neural network for automated detection of myocardial infarction using ECG signals. Inf. Sci..

[17] Cao Y., Liu W., Zhang S., Xu L., Zhu B., Cui H., Geng N., Greenwald S.E. (2022). Detection and localization of myocardial infarction based on multi-scale resnet and attention mechanism. Front. Physiol..

[18] Jun T.J., Nguyen H.M., Kang D., Kim D., Kim D., Kim Y.H. (2018). ECG arrhythmia classification using a 2-D convolutional neural network. arXiv.

[19] Goldberger A.L., Amaral L.A., Glass L., Hausdorff J.M., Ivanov P.C., Mark R.G., Stanley H.E., Mietus J.E., Moody G.B., Peng C.K. (2000). PhysioBank, PhysioToolkit, and Physionet: Components of a new research resource for complex physiologic signals. Circulation.

[20] National Institute of General Medical Sciences and National Institute of Biomedical Imaging and Bioengineering, PhysioBank. https://physionet.org/physiobank/.

[21] Gu J., Wang Z., Kuen J., Ma L., Shahroudy A., Shuai B., Chen T., Liu T., Wang G., Wang X. (2018). Recent advances in convolutional neural networks. Pattern Recognit..

[22] Krizhevsky A., Sutskever I., Hinton G.E. Imagenet classification with deep convolutional neural networks. Proceedings of the Neural Information Processing Systems.

[23] Boureau Y.L., Ponce J., LeCun Y. A theoretical analysis of feature pooling in visual recognition. Proceedings of the 27th International Conference on Machine Learning (ICML-10).

[24] LeCun Y., Haffner P., Bottou L., Bengio Y. (1999). Object recognition with gradient-based learning. Lect. Notes Comput. Sci..

[25] Dong S., Wang P., Abbas K. (2021). A survey on deep learning and its applications. Comput. Sci. Rev..

[26] Liu W., Huang Q., Chang S., Wang H., He J. (2018). Multiple-feature-branch convolutional neural network for myocardial infarction diagnosis using electrocardiogram. Biomed. Signal Process. Control.

[27] Remez T., Litany O., Giryes R., Bronstein A.M. (2018). Class-aware fully convolutional Gaussian and Poisson denoising. IEEE Trans. Image Process..

[28] Wang F., Jiang M., Qian C., Yang S., Li C., Zhang H., Wang X., Tang X. Residual attention network for image classification. Proceedings of the IEEE Conference on Computer Vision and Pattern Recognition.

[29] Pires R.G., Santos D.F., Santos C.F., Santana M.C., Papa J.P. (2021). Image denoising using attention-residual convolutional neural networks. arXiv.

[30] Fu L., Lu B., Nie B., Peng Z., Liu H., Pi X. (2020). Hybrid network with attention mechanism for detection and location of myocardial infarction based on 12-lead Electrocardiogram signals. Sensors.

[31] Han C., Shi L. (2019). MLResNet: A novel network to detect and locate myocardial infarction using 12 leads ECG. Comput. Methods Programs Biomed..

[32] Selvaraju R.R., Cogswell M., Das A., Vedantam R., Parikh D., Batra D. Grad-cam: Visual explanations from deep networks via gradient-based localization. Proceedings of the IEEE International Conference on Computer Vision.

[33] Hayıroğlu M.İ., Çınar T., Çiçek V., Asal S., Kılıç Ş., Keser N., Orhan A.L., Uzun M. (2021). A simple formula to predict echocardiographic diastolic dysfunction—electrocardiographic diastolic index. Herz.

[34] Peng P., You M., Xu W., Li J. (2021). Fully integer-based quantization for mobile convolutional neural network inference. Neurocomputing.

[35] Choudhary T., Mishra V., Goswami A., Sarangapani J. (2022). Inference-aware convolutional neural network pruning. Future Gener. Comput. Syst..

